# The Cell Adhesion Activity of the Joining Peptide of Proopiomelanocortin

**DOI:** 10.3390/molecules28237754

**Published:** 2023-11-24

**Authors:** Kyona Hiroshima, Nana Sakata, Tadafumi Konogami, Shigeru Shimamoto, Yuji Hidaka

**Affiliations:** Faculty of Science and Engineering, Kindai University, 3-4-1 Kowakae, Higashi-Osaka, Osaka 577-8502, Japan

**Keywords:** ACTH, cell adhesion, integrin, joining peptide, peptide hormone, POMC

## Abstract

Proopiomelanocortin (POMC) is a precursor protein of several peptide hormones, such as ACTH and β-endorphin. Almost all of the peptide hormones in POMC have been drastically investigated in terms of their biological activities. However, the biological activity of the joining peptide region (JP) in POMC is unknown. Therefore, to explore the biological activity of JP, sequence analyses of mammalian POMC were performed. We found an -Arg-Gly-Asp- (RGD) motif in several mammalian species, such as porcine, suggesting that JP has cell adhesion activity. To validate this hypothesis, the cell adhesion activities of the synthetic porcine JP peptides were examined using 293T cells. Cell adhesions were observed in a concentration-dependent manner of the JP peptides. In addition, the JP peptide competitively inhibited cell adhesion to the POMC-coated plates. Moreover, the cell adhesion activity of the joining peptide was inhibited by the addition of EDTA, indicating that the JP peptide mediates the cell adhesion activity via a receptor protein, integrin. Interestingly, a human JP peptide, which possesses an -Arg-Ser-Asp- (RSD) sequence in place of the RGD sequence, exhibited a higher ability in the cell adhesion activity than that of the porcine JP peptide, suggesting that the cell adhesion activity of the joining peptide is developed during the molecular evolution of POMC. In conclusion, our results reveal that the joining peptide in POMC plays an important role during cell adhesion and provide useful information related to signal transduction of nerve peptide hormones derived from POMC.

## 1. Introduction

Peptide hormones play a crucial role as the main substances responsible for the transmission of information in the endocrine system and significantly contribute to the maintenance of homeostasis. Generally, many endocrine and neuroendocrine peptides are translated as relatively large precursor proteins, pre-proproteins. The pre-region, known as the signal sequence, is cleaved upon translocation into the endoplasmic reticulum. The precursor proteins form a three-dimensional structure in the endoplasmic reticulum, which is closely related to the processing of the precursor, as it undergoes specific cleavage by enzymes, such as prohormone-converting enzymes, during the maturation process.

Proopiomelanocortin (POMC) is one of the most well-known precursor proteins of peptide hormones [1]. POMC contains various peptide hormones, such as adrenocorticotropic hormone (ACTH), β-endorphin, and melanocyte-stimulating hormones (MSHs), within its molecule (Supplementary Materials Figure S1).

POMC is initially synthesized as a pre-POMC protein, which contains a signal peptide consisting of 26 residues, and the signal sequence is cleaved during translocation to the endoplasmic reticulum [2]. Subsequently, in the Golgi apparatus, POMC undergoes glycosylation to become mature POMC, which is then inserted into secretory granules. Furthermore, POMC undergoes tissue-specific processing primarily by two types of prohormone convertases (PC; endoproteolytic enzymes) [1,3]. First, in the anterior pituitary, POMC is cleaved by PC1 to produce N-POMC, ACTH, and β-lipotropin (β-LPH), as shown in Supplementary Materials Figure S2. Then, N-POMC is cleaved by PC2 to produce an N-terminal fragment including γ-MSH and a joining peptide region (JP). The POMC fragment peptides were further cleaved by PC2 to produce small peptide hormones in the intermediate lobe of the pituitary (Supplementary Materials Figure S2).

**Figure 1:**
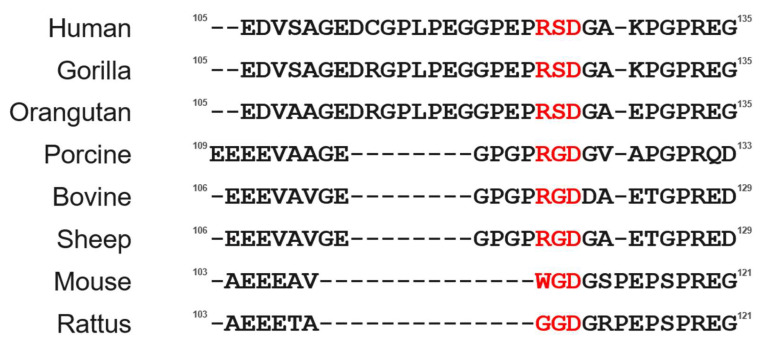
Amino acid sequences of several kinds of mammalian POMC. The putative cell adhesion sites are indicated by red.

Considerable knowledge has been amassed concerning the generation process and functions of bioactive peptides derived from POMC [1,4]. However, the biological activity of the joining peptide region (JP) remains unknown, despite extensive research on POMC-derived hormones [1,5,6]. Therefore, in this study, to explore the biological activity of the JP peptide of POMC, we performed sequence analyses. As a result, we identified that the JP peptide derived from porcine POMC contains an -Arg-Gly-Asp- (RGD) sequence, which is a common motif for cell adhesion ability (Figure 1) [7,8,9,10,11]. Thus, we demonstrate cell adhesion activity of the chemically synthesized porcine JP peptides. In addition, we observed higher cell adhesion activity of the human JP peptide, which contains an -Arg-Ser-Asp- (RSD) sequence in place of the RGD sequence. Based on these results, we concluded that cell adhesion activity is an intrinsic function of the JP peptide of POMC. Therefore, we performed the cell adhesion activity of the synthetic joining peptide using HEK293T cells as a model of adhesive cells.

## 2. Results and Discussion

### 2.1. Characterization of the JP Peptide of POMC

The sequence alignments of the JP peptides revealed significant variations among mammalian species, as shown in Figure 1. However, we found that several mammalian species of POMC, such as porcine and bovine, provide the -Arg-Gly-Asp- (RGD) sequence, which is well known as the cell adhesion motif and acts as a specific binding site for cell adhesion proteins, such as an integrin family. Although the sequence is not conserved in all mammalian species, these facts prompted us to hypothesize that cell adhesion activity is an intrinsic function of JP.

**Figure 2:**
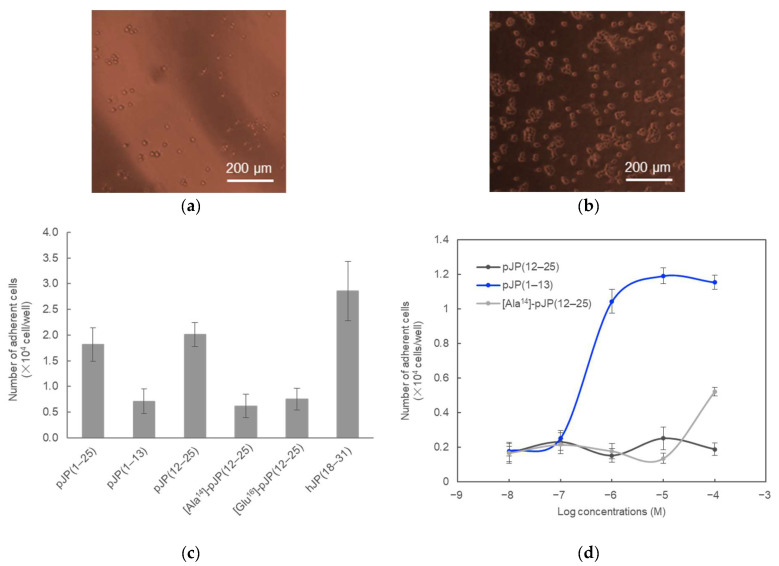
The cell adhesion activities of the synthetic JP fragments using the pJP(1–13)- or pJP(12–25)-coated plates in (**a**) and (**b**), respectively. Numbers of adherent cells are summarized in (**c**). Error bars: ±1 standard deviation (*n* = 3). 293T cells were incubated in the presence of several concentrations of the pJP(1–13), pJP(12–25), or [Ala^14^]-pJP(12–25) peptides (black, blue and gray lines, respectively) (**d**). Error bars: ±1 standard deviation (*n* = 3).

To validate our hypothesis, we synthesized several types of porcine JP peptides, such as pJP(1–13) and pJP(12–25), and performed cell adhesion experiments using HEK 293T cells, as shown in Figure 2. As expected, the pJP(1–25) and pJP(12–25) peptides, carrying the RGD motif, exhibited cell adhesion activity, whereas there were no significant cell adhesion activities for the pJP(1–13), [Ala^14^]-pJP(12–25) and [Glu^16^]-pJP(12–25) peptides, as a negative control [12,13,14]. The cell adhesion activity of pJP(12–25) peptide was also confirmed using PC12 cells. In addition, the number of adherent cells increased in a concentration-dependent manner with pJP(12–25), as shown in Figure 2d. These results indicate that the JP peptide has cell adhesion activity and that its function depends on the RGD sequence.

Thus, we determined the cell adhesion activity of the porcine joining peptide. However, the RGD sequence of the JP peptide is not conserved in all mammalian species, especially in humans. The JP peptide in human POMC has an -Arg-Ser-Asp- (RSD) sequence instead of the -Arg-Gly-Asp- (RGD) sequence in porcine POMC, as shown in Figure 1. Therefore, to further investigate whether cell adhesion ability is a common function of the JP peptide in mammalian species, we also performed a cell adhesion assay of the human JP peptide, hJP(18–31). The synthetic hJP(18–31) peptide exhibited cell adhesion activity, as shown in Figure 2. Interestingly, the hJP(18–31) peptide showed slightly higher activity than the pJP(12–25) peptide. The RSD sequence of the JP peptide likely possesses a higher ability for cell adhesion and is conserved in primates. We propose that the RSD sequence is naturally employed as the cell adhesion site during the molecular evolution of the JP peptide. Indeed, the RSD sequence was artificially found as a cell adhesion moiety of tendamistat, an inhibitor of α-amylase, using the phage display system [7]. Structure-activity relationships of the JP peptide, including other species, regarding molecular evolution are in progress in our laboratory.

### 2.2. Preparation of the POMC

Based on our finding that the JP peptides provide cell adhesion activity, it is possible to speculate that the POMC protein itself may also have a cell adhesion function. In addition, it is also interesting to obtain structural information regarding the processing mechanism of POMC. Therefore, to obtain structural information and evaluate the cell-binding activity of POMC, we established a high-level expression system in *E. coli* cells and purified the recombinant POMC protein.

**Figure 3:**
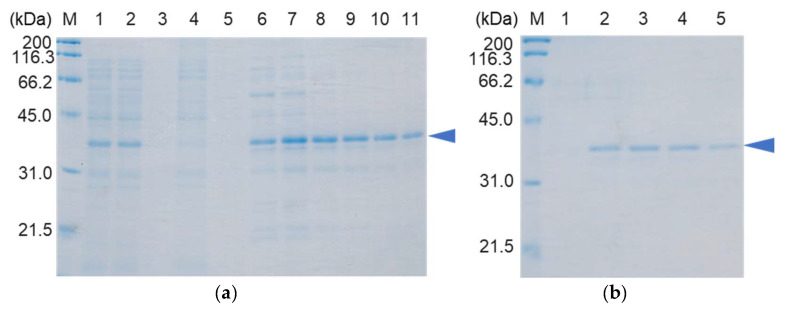
SDS-PAGE of the Gly-POMC-His_6_ protein purified by the Ni-affinity (**a**) and hydroxyapatite chromatography (**b**). (**a**) Lane 1, total cells; lanes 2 and 3, supernatants and precipitates after centrifugation of the sonicated cells, respectively; lanes 4 and 5, non-bound and washed fractions, respectively; lanes 6–11, fractions of 400 mM imidazole solutions. (**b**) Lanes 1–5, fractions of 200 mM sodium phosphate buffer (pH 6.0). M represents marker proteins. The Gly-POMC-His_6_ protein are indicated by arrow heads.

Our preliminary experiments for the expression of the intact form of porcine POMC using a combination of the pET-17b vector and an *E. coli* expression system were unsuccessful. Therefore, to efficiently produce recombinant POMC in *E. coli* cells, the cDNA of proopiomelanocortin with additional cDNA encoding an N-terminal Gly residue and a C-terminal His_6_-tag sequence, in which the codon bias was optimized for the *E. coli* expression system, was chemically prepared. POMC has two conserved disulfide bonds in its N-terminal region [15,16], and these disulfide bonds play an important role in the sorting of POMC into secretory granules [17]. Therefore, SHuffle T7 Express *E. coli* cells, which constitutively express the disulfide bond isomerase DsbC and are suitable for protein expression with multiple disulfide bonds, were adopted for overexpressing the recombinant POMC protein. As shown in Figure 3, the recombinant POMC protein, Met-Gly-POMC-His_6_ (NGCH-POMC), was successfully obtained as a soluble form. The degradation products of POMC were also observed in the protein solution, which was purified by NAC. Therefore, the protein was further purified by HAC, and the purified POMC was homogeneously obtained (approximately 5 mg from 1 L of culture cells) and confirmed by SDS-PAGE (Figure 3).

### 2.3. Cell-Binding Activity of POMCs

**Figure 4:**
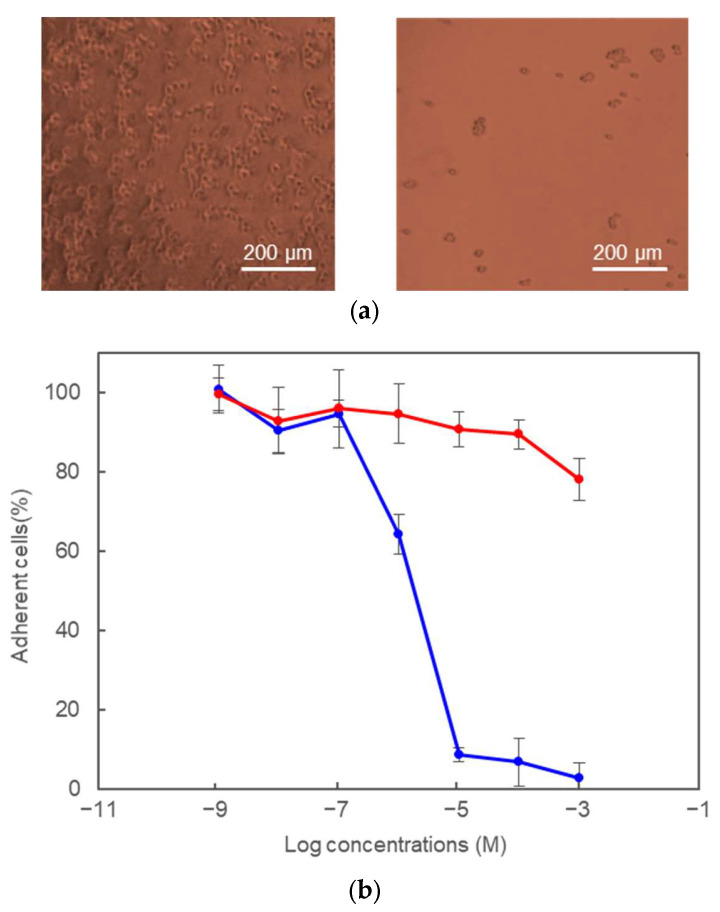
The cell adhesion activities of POMC (**a**) and inhibition assay by the JP peptides (**b**). (**a**) 293T cells were incubated in wells using the POMC- (**left**) or lysozyme- (**right**) coating plates, respectively. (**b**) 293T cells were incubated in wells using the POMC-coating plated after the incubation in the presence of several concentrations of the pJP(12–25) or [Ala^14^]-pJP(12–25) peptides (blue and red lines, respectively). Error bars: ±1 standard deviation (*n* = 3).

It has been reported that POMC is associated with the secretory granule membrane before processing in frog and mouse pars intermedia cells [18] using POMC-expressing cells. To investigate the cell-binding activity of POMC at the molecular level, we performed a cell adhesion experiment using the purified recombinant POMC protein. The recombinant POMC protein clearly increased 293T cell adhesion on the plates, as shown in Figure 4a. In addition, the number of adherent cells increased in the concentration-dependent manner of the recombinant POMC protein, as shown in Supplementary Materials Figure S3. These results were consistent with those of the JP peptides in this study and suggested that POMC interacts with a certain protein present in/on the membrane.

**Figure 5:**
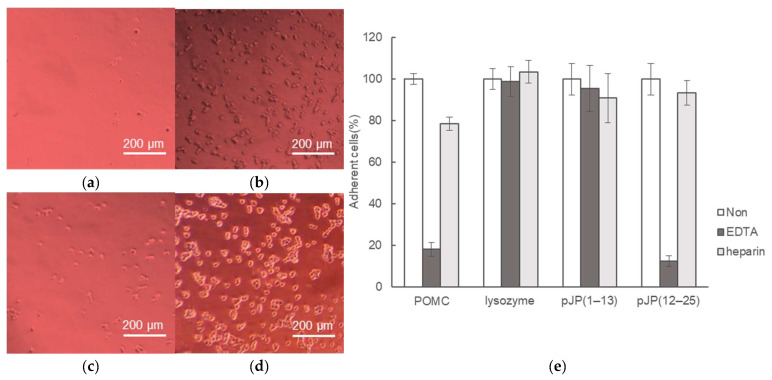
Inhibition assay of the cell adhesion activities of the POMC protein (**a**,**b**) and the JP peptide (**c**,**d**) by EDTA and heparin. 293T cells were incubated in wells using the POMC- or the pJP(12–25)-coating plate in the presence of EDTA (**a**,**c**) or heparin (**b**,**d**). The ratio of the numbers of the attached cells to total cells are depicted in (**e**). Error bars: ±1 standard deviation (*n* = 3).

To further investigate whether the cell-binding activity of POMCs is dependent on the RGD motif, we performed a competitive assay of the cell adhesion activities of pJP(12–25) and [Ala^14^]-pJP(12–25), as shown in Figure 4b. The pJP(12–25) peptide competitively exhibited the cell adhesion activity of the POMC protein in a concentration-dependent manner, whereas [Ala^14^]-pJP(12–25) did not inhibit the cell-binding activity of the POMC protein, revealing that the cell-binding activity of POMC is dependent on the RGD sequence in the JP peptide. Furthermore, the cell-binding activities of the POMC protein and the pJP(12–25) peptide were inhibited by the addition of EDTA but not heparin (Figure 5). It is well known that cell adhesion via integrin proteins, is inhibited by EDTA. Indeed, integrin proteins play an important role of cell adhesion of 293T cells [19]. Therefore, our results indicate that the joining peptide of POMC is recognized by the integrin protein(s) on the cell membrane.

### 2.4. Conformational Analyses of the JP Peptides and the POMC Protein by CD Spectroscopy

**Figure 6:**
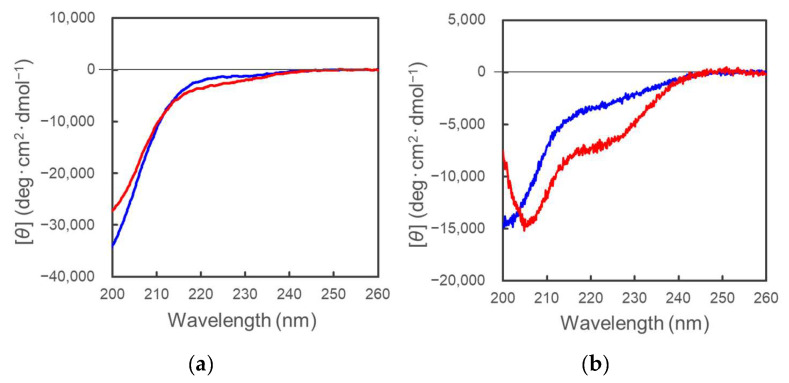
CD spectra of the pJP(1–25) peptide (**a**) and the POMC protein (**b**). CD measurements were carried out in the presence (red) or absence (blue) of 20% trifluoroethanol (TFE).

The JP peptides possess cell-binding activity at the RGD or RSD sites by interacting with the integrin protein(s). The β- or γ-turn structures of the motif RGD are suitable for integrin binding [20,21,22]. To obtain structural information related to the cell-binding activities of the JP peptides, CD measurements were performed. The JP peptides did not exhibit the typical CD spectra for the β-turn conformation, suggesting that the JP peptides have a flexible conformation without binding to the integrin protein(s) (Figure 6a).

Furthermore, to characterize the overall structure of the POMC molecule, secondary structural analyses were performed by collecting CD spectra. As shown in Figure 6b, POMC did not appear to adopt any specific conformation, such as an intrinsically disordered protein.

To obtain further structural information on the JP peptides and POMC, CD measurements were performed in the presence or absence of trifluoroethanol (TFE). TFE can provides a hydrophobic environment and induces α-helix formation in a protein that has the inherent capability to adopt an α-helix conformation [23]. As shown in Figure 6, α-helix formation was induced for the POMC protein in the presence of 20% TFE but not for the JP peptides, suggesting that POMC may possess an α-helical structure under hydrophobic conditions but not the joining peptide region. This conformational change in the POMC molecule may be induced by interaction with hydrophobic membranes.

In this study, we focused on the cell adhesion activity of the JP region of POMC in mammals. We clearly showed that the JP peptides carrying the RGD or RSD sequences have cell-binding activity. In addition, we also determined that JP peptides function as cell adhesion factors by interacting with integrin protein(s). Furthermore, we successfully obtained a sufficient amount of POMC to evaluate its cell adhesion activity. The recombinant POMC protein was expressed as a soluble form and homogeneously purified by a combination of several types of chromatography. The recombinant POMC clearly showed cell-binding activity by the RGD motif, although the protein does not have a characteristic structure by itself, like an intrinsically disordered protein. The binding activity of POMC to the cell membrane (protein) is reasonable for efficient cleavage because the major prohormone convertases, PC1 and PC2, involved in the cleavage of POMC, are membrane proteins [3]. It is thought that tissue-specific cleavage by PC2 is controlled by pH and the presence of cofactors [1]. However, there is not much discussion about the regulation mechanism for stepwise cleavages by PC1. Thus, we speculate that the α helical conformation of the POMC molecule when interacting with the membrane (protein) is involved in the regulation of stepwise cleavage by PC1.

In conclusion, we clearly demonstrated the cell adhesion activity of the joining peptide region of POMC. In addition, we established an *E. coli* expression system of the POMC protein. Our convenient preparation system for POMC protein will further accelerate the investigation of the biological activity of POMC and its fragment hormones. Our findings provide new insight into the role of the joining peptide region of POMC.

## 3. Materials and Methods

### 3.1. Materials

Boc-amino acids, hexafluorophosphate benzotriazole tetramethyl uranium (HBTU), and 1-hydroxybenzotriazole (HOBt) were purchased from the Peptide Institute, Inc. (Osaka, Japan) and Watanabe Chemical Industries, Ltd. (Hiroshima, Japan). All chemicals and solvents used were of reagent grade. The cDNA encoding POMC used in this study (Supplementary Materials Figure S4) was prepared by Hokkaido System Science CO, Ltd. (Hokkaido, Japan).

### 3.2. Construction of Expression Vectors of Recombinant POMC in E. coli Cells

The cDNA of POMC was prepared by PCR using the synthetic cDNA encoding POMC (Supplementary Materials Figure S4) as a template. PCR was performed using Plutinum Pfx DNA polymerase (Invitrogen; Thermo Fisher Scientific, Inc., Santa Clara, CA, USA). The amplified cDNA was then subcloned into the pET-17b expression vector (Novagen, Glendale, CA, USA) with an N-terminal Gly and a C-terminal 6 × His tag, following the introduction of a *Nde*I and an *Xho*I site at its 5′ and 3′ end, respectively. The insertion of the Gly residue at the N-terminus of POMC effectively suppressed the degradation of POMC during expression in *E. coli*. The resulting expression vector, referred to as pNGCH-POMC, contained the cDNA of Gly-POMC-His_6_. The cDNA sequence of the vector was confirmed by the Eurofins Japan DNA sequencing service (Tokyo, Japan).

### 3.3. Protein Expression and Purification of Recombinant POMC

SHuffle T7 Express *E. coli* cells (New England Biolabs Japan Inc., Tokyo, Japan), transformed with the expression vector (pNGCH-POMC), were incubated at 37 °C in 2 × YT medium (1 L) supplemented with ampicillin (50 mg/L). The production of mutant proteins was induced by the addition of isopropyl β-thiogalactopyranoside (final concentration, 1 mM) at the mid-log phase. After another 3 h of incubation at 37 °C, cells were harvested by centrifugation (4 °C, 5000× *g*, 15 min). The cells were resuspended in 50 mM sodium phosphate buffer (pH 6.0) containing 5% glycerol and 1 mM phenylmethylsulfonyl fluoride and sonicated at 4 °C. The bacterial lysate was clarified by centrifugation at 20,000× *g* for 30 min at 4 °C.

The recombinant protein was purified by Ni affinity chromatography (NAC) using ECONO GRADIENT POMP (Bio-Rad Laboratories, Inc., Hercules, CA, USA), according to the manufacturer’s protocol with small modifications. Briefly, the protein solution was applied to a Ni Sepharose Fast Flow column (5 mL, GE healthcare Japan, Tokyo, Japan) that had been equilibrated with NAC buffer I (50 mM phosphate buffer containing 500 mM NaCl, 5% glycerol, and 50 mM imidazole; pH 6.0) and eluted with NAC buffer II (50 mM phosphate buffer 5% glycerol, and 400 mM imidazole; pH 6.0). The concentrations of the eluted proteins were determined by SDS-PAGE.

The target protein was further purified by hydroxyapatite chromatography (HAC), according to the standard protocol [24] with small modifications. Briefly, the protein solution was applied to 5 mL of Fast Flow-Type hydroxyapatite resins (100–200 mesh, nacalai tesque Inc., Kyoto, Japan) that had been equilibrated with HAC buffer I (50 mM phosphate buffer containing 5% glycerol, pH 6.0). The column was subsequently washed with HAC buffer I, HAC buffer II (100 mM phosphate buffer containing 5% glycerol, pH 6.0), and HAC buffer III (150 mM phosphate buffer containing 5% glycerol, pH 6.0). The protein was then eluted with HAC buffer IV (200 mM phosphate buffer containing 5% glycerol, pH 6.0). The concentrations of the purified proteins were determined by SDS-PAGE or MALDI-TOF/MS analyses.

### 3.4. Peptide Synthesis

The JP peptides were chemically synthesized on Merrifield’s resin using HBTU/HOBt as the condensation reagent by the Boc solid phase method [25,26,27]. The protected peptide resins were treated with anhydrous hydrogen fluoride to remove the protecting groups and the resin. The target peptides were extracted with 10% AcOH, as previously reported [25,27]. The synthetic peptides were purified by HPLC, as described below, and characterized by amino acid and mass spectrometric analyses, as summarized in Table 1.

### 3.5. Reversed-Phase High-Performance Liquid Chromatography (RP-HPLC)

The HPLC apparatus was composed of a Waters M600 multisolvent delivery system (Bedford, MA, USA) equipped with a Hitachi L-3000 detector and a D-2500 chromato-integrator. Peptides were separated by RP-HPLC using an Inertsil ODS-4 column (4.6 × 150 mm, GL Sciences Inc., Tokyo, Japan) and confirmed by MALDI-TOF/MS analyses, as described below.

### 3.6. Matrix-Assisted Laser Desorption/Ionization Time-of-Flight Mass Spectrometry (MALDI-TOF/MS)

The molecular weights of proteins were determined using an AXIMA confidence spectrometer (SHIMADZU Co., Kyoto, Japan) in the positive-ion mode. Mass spectrometric analyses of proteins and peptides were performed in the linear or reflector modes using 3,5-dimethoxy-4-hydroxycinnamic acid (Tokyo Chemical Industry Co., Ltd., Tokyo, Japan), and α-cyano-4-hydroxycinnamic acid (Sigma-Aldrich Co., Tokyo, Japan) as matrices, respectively. In a typical run, the lyophilized sample (ca. 0.1 nmol) was dissolved in 0.05% TFA aq/50% CH_3_CN (1 μL), mixed with 1 μL of a matrix solution (10 mg/mL), and air-dried on the sample plate for use in MALDI-TOF/MS, as previously reported [25,28]. The mass values of the peptides observed by MALDI-TOF/MS are summarized in Table 1.

### 3.7. CD Measurements

The circular dichroism (CD) spectra were recorded on a JASCO J720 spectrometer (JASCO Corporation, Tokyo, Japan) at 25 °C. The HPLC-purified peptides were dissolved in 50 mM sodium phosphate buffer (pH 7.0), whereas the POMC protein that had been purified by hydroxyapatite chromatography was dialyzed against 50 mM sodium phosphate buffer (pH 7.0) in the presence or absence of 20% trifluoroethanol (TFE).

### 3.8. Cell Adhesion Assay

Multiwell 24-well plates (Corning Inc., New York, NY, USA) were coated with purified POMC protein or JP peptides (300 μL of 10^−8^ to 10^−4^ M peptide or protein solutions for each well) at room temperature overnight in 100 mM sodium phosphate buffer (pH 7.0). Then, the solutions were removed and the wells were washed with 500 μL of PBS [29].

HEK 293T (human embryonic kidney cells 293T) cells were grown and maintained in DMEM with 10% fetal bovine serum (FBS) in a 6 cm dish [25]. The cells were incubated at 37 °C for 3 days in a 5% CO_2_ incubator, harvested with PBS (1 mL), and divided into each well (4 × 10^5^ cell/well) of a 24-well plate, which was pre-coated with the peptide or protein solutions. The cells were incubated for 1 h at 37 °C in a 5% CO_2_ incubator and observed in a counting chamber (Erma, Tokyo, Japan). In addition, to investigate the inhibition of the cell adhesion activities by EDTA and heparin, the cells were incubated for 1 h at 37 °C in a 5% CO_2_ incubator in the presence of 5 mM EDTA or 10 mg/mL heparin, and observed in a counting chamber. Cells were counted from 4 random microscope fields of a cell counting chamber slide for each sample in 3 independent experiments.

### 3.9. Competitive Binding Assay

Multiwell 24-well plates (Corning Inc., New York, NY, USA) were coated with purified POMC protein (300 μL of 10^−6^ M protein solution for each well) at room temperature overnight in 100 mM sodium phosphate buffer (pH 7.0).

HEK 293T cells were grown and maintained in DMEM with 10% fetal bovine serum (FBS) in a 6 cm dish. The cells were incubated at 37 °C for 3 days in a 5% CO_2_ incubator and were harvested with PBS (500 μL) and then divided into small portions (100 μL). The aliquots were recentrifuged at 3500 rpm for 10 min at 4 °C and resuspended/mixed with various concentrations of the JP peptide solution (10^−9^–10^−3^ M) in DMEM (200 μL) at 37 °C for 45 min. The reaction mixtures were transferred to the POMC-coated wells in a 24-well plate, incubated at 37 °C for 1 h in a 5% CO_2_ incubator, and observed in a counting chamber. Cells were counted from 4 random microscope fields of a cell counting chamber slide for each sample in 3 independent experiments.

## Figures and Tables

**Table 1:** The mass values of the synthetic peptides determined by MALDI-TOF/MS.

Peptide Names	Amino Acid Sequences	[M + H]^+^_calc._	[M + H]^+^_obs._
pJP	^1^EEEEVAAGEGPGPRGDGVAPGPRQD^25^ *	2475.1	2476.5
pJP(1–13)	^1^EEEEVAAGEGPGP^13^	1291.6 **	1291.3
pJP(12–25)	^12^GPRGDGVAPGPRQD^25^	1377.7	1377.1
[Ala^14^]-pJP(12–25)	^12^GPAGDGVAPGPRQD^25^	1292.6	1295.4
[Glu^16^]-pJP(12–25)	^12^GPRGEGVAPGPRQD^25^	1391.7	1392.0
hJP(18–31)	^18^EPRSDGAKPGPREG^31^	1451.7	1451.4

* The cell adhesion motif (RGD) is underlined. ** The *m*/*z* values are indicated by [M + Na]^+^.

## Data Availability

Data are contained within the article and Supplementary Materials.

## Supplementary Materials

The following supporting information can be downloaded at: https://www.mdpi.com/article/10.3390/molecules28237754/s1, Figure S1: amino acid sequence of porcine POMC and bioactive peptides included. Disulfide bonds are indicated by solid blue lines; Figure S2: schematic drawing of the processing of porcine POMC in the anterior pituitary and the intermediate lobe of the pituitary; Figure S3: 293T cells were incubated in the presence of several concentrations of peptide and proteins; Figure S4: the cDNA sequence of porcine POMC used in this study.

## Abbreviations

ACTH, adrenocorticotropic hormone; Boc, *tert*-butoxycarbonyl; CD, circular dichroism; DMEM, Dulbecco’s modified Eagle medium; EDTA, ethylenediamine-*N*,*N*,*N*′,*N*′-tetraacetic acid; FBS, fetal bovine serum; HAC, hydroxyapatite chromatography; JP, joining peptide region; MALDI-TOF/MS, matrix-assisted laser desorption/ionization time-of-flight mass spectrometry; MSHs, melanocyte-stimulating hormones; NAC, Ni affinity chromatography; PC, prohormone convertase; PMSF, phenylmethylsulfonyl fluoride; POMC, proopiomelanocortin; RGD, -Arg-Gly-Asp-; RP-HPLC, reversed-phase high performance liquid chromatography; RSD, -Arg-Ser-Asp-; TFE, trifluoroethanol; β-LPH, β-lipotropin.
