# Supplementary material for: The Cell Adhesion Activity of the Joining Peptide of Proopiomelanocortin
Source: Molecules. 2023 Nov 24;28(23):7754. doi: 10.3390/molecules28237754 (PMC10707936; doi:10.3390/molecules28237754)
Supplement: Supplementary file 1 [file molecules-28-07754-s001.zip › molecules-2694203-supplementary.pdf]

Supplementary Materials

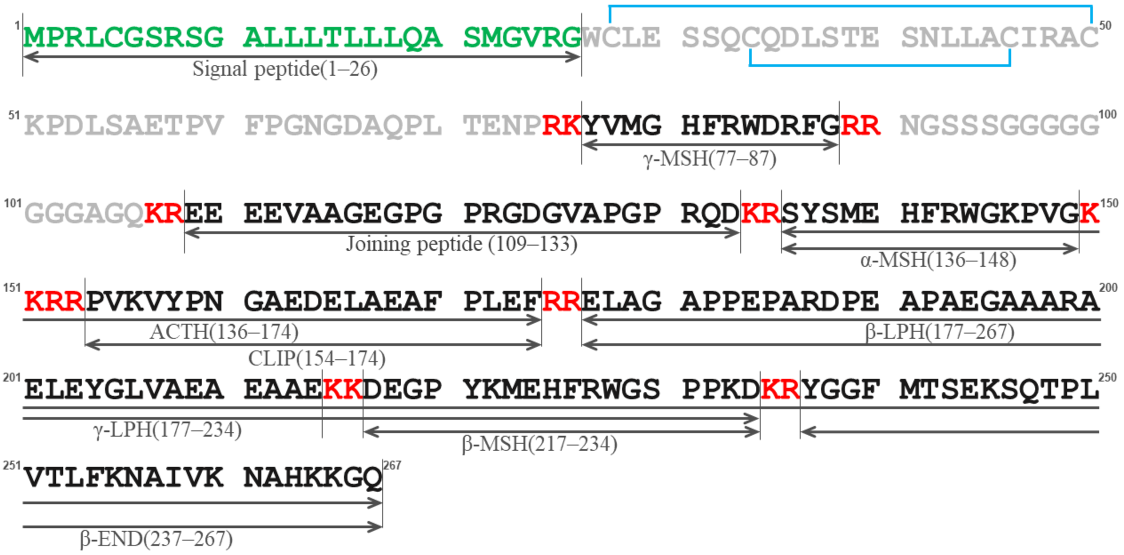

Figure S1. Amino acid sequence of porcine POMC and bioactive peptides included. Disulfide bonds are indicated by solid blue lines.

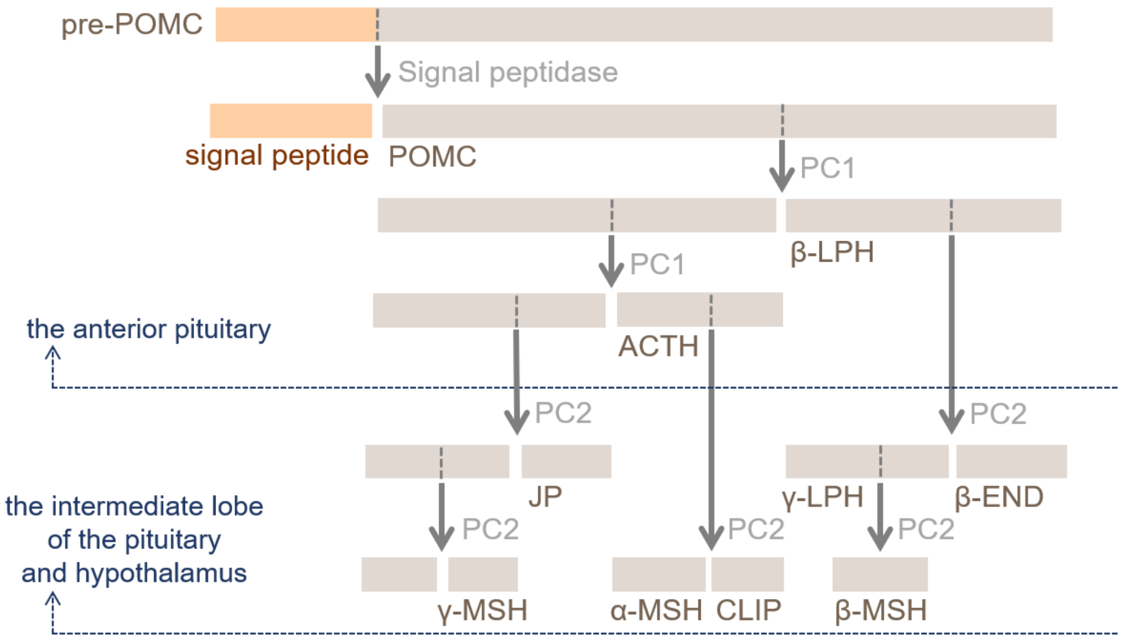

Figure S2. Schematic drawing of the processing of porcine POMC in the anterior pituitary and the intermediate lobe of the pituitary.

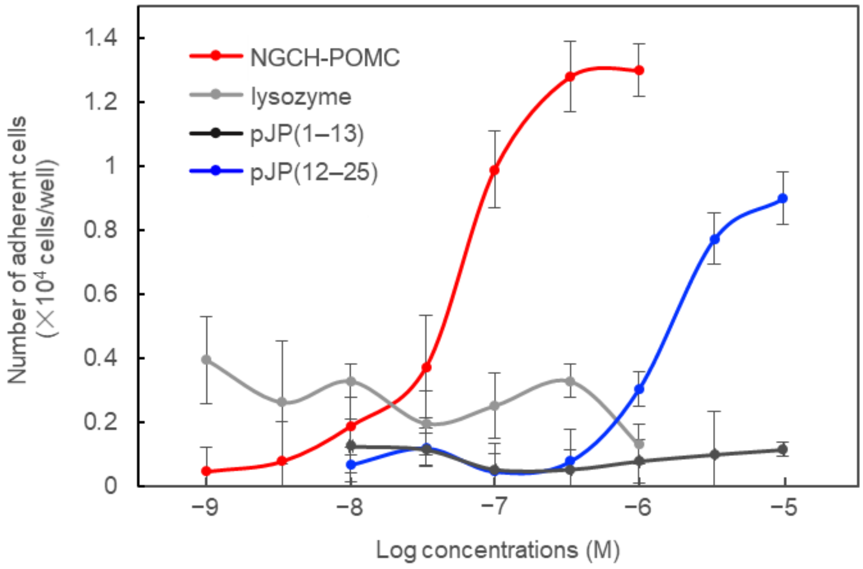

Figure S3. 293T cells were incubated in the presence of several concentrations of peptide and proteins. Error bars: ± 1 standard deviation ($n = 3$)

```
1          10          20          30          40          50          60
TGG TGC CTG GAA TCC TCC CAA TGT CAA GAC CTG TCA ACC GAA AGT AAT CTG CTG GCC TGT ATC CGT GCG
--- --- --- --- --- --- --- --- --- --- --- --- --- --- --- --- --- --- --- --- --- --- ---
Trp Cys Leu Glu Ser Ser Gln Cys Gln Asp Leu Ser Thr Glu Ser Asn Leu Leu Ala Cys Ile Arg Ala

70          80          90          100         110         120         130
TGT AAA CCG GAC CTG AGC GCT GAA ACC CCG GTT TTT CCG GGT AAC GGT GAT GCA CAG CCG CTG ACG GAA
--- --- --- --- --- --- --- --- --- --- --- --- --- --- --- --- --- --- --- --- --- --- ---
Cys Lys Pro Asp Leu Ser Ala Glu Thr Pro Val Phe Pro Gly Asn Gly Asp Ala Gln Pro Leu Thr Glu

140         150         160         170         180         190         200
AAT CCG CGT AAA TAT GTC ATG GGT CAT TTT CGT TGG GAC CGC TTC GGT CGT CGC AAC GGC AGC TCT AGT
--- --- --- --- --- --- --- --- --- --- --- --- --- --- --- --- --- --- --- --- --- --- ---
Asn Pro Arg Lys Tyr Val Met Gly His Phe Arg Trp Asp Arg Phe Gly Arg Arg Asn Gly Ser Ser Ser

 210         220         230         240         250         260         270
GGC GGT GGC GGT GGC GGT GGC GGT GCT GGT CAG AAA CGT GAA GAA GAA GAA GTG GCA GCA GGT GAA GGT
--- --- --- --- --- --- --- --- --- --- --- --- --- --- --- --- --- --- --- --- --- --- ---
Gly Gly Gly Gly Gly Gly Gly Gly Ala Gly Gln Lys Arg Glu Glu Glu Glu Val Ala Ala Gly Glu Gly

   280         290         300         310         320         330         340
CCG GGT CCG CGC GGT GAT GGT GTG GCA CCG GGT CCG CGT CAA GAC AAA CGC TCC TAT TCA ATG GAA CAC
--- --- --- --- --- --- --- --- --- --- --- --- --- --- --- --- --- --- --- --- --- --- ---
Pro Gly Pro Arg Gly Asp Gly Val Ala Pro Gly Pro Arg Gln Asp Lys Arg Ser Tyr Ser Met Glu His

    350         360         370         380         390         400         410
TTC CGC TGG GGT AAA CCG GTG GGC AAA AAA CGT CGC CCG GTG AAA GTT TAC CCG AAT GGT GCC GAA GAT
--- --- --- --- --- --- --- --- --- --- --- --- --- --- --- --- --- --- --- --- --- --- ---
Phe Arg Trp Gly Lys Pro Val Gly Lys Lys Arg Arg Pro Val Lys Val Tyr Pro Asn Gly Ala Glu Asp

     420         430         440         450         460         470         480
GAA CTG GCT GAA GCG TTT CCG CTG GAA TTT CGT CGC GAA CTG GCA GGT GCA CCG CCG GAA CCG GCC CGT
--- --- --- --- --- --- --- --- --- --- --- --- --- --- --- --- --- --- --- --- --- --- ---
Glu Leu Ala Glu Ala Phe Pro Leu Glu Phe Arg Arg Glu Leu Ala Gly Ala Pro Pro Glu Pro Ala Arg

       490         500         510         520         530         540         550
GAT CCG GAA GCT CCG GCA GAA GGT GCA GCT GCG CGC GCA GAA CTG GAA TAC GGC CTG GTT GCC GAA GCA
--- --- --- --- --- --- --- --- --- --- --- --- --- --- --- --- --- --- --- --- --- --- ---
Asp Pro Glu Ala Pro Ala Glu Gly Ala Ala Ala Arg Ala Glu Leu Glu Tyr Gly Leu Val Ala Glu Ala

        560         570         580         590         600         610         620
GAA GCC GCA GAG AAA AAA GAT GAA GGT CCG TAT AAA ATG GAA CAT TTT CGT TGG GGC AGT CCG CCG AAA
--- --- --- --- --- --- --- --- --- --- --- --- --- --- --- --- --- --- --- --- --- --- ---
Glu Ala Ala Glu Lys Lys Asp Glu Gly Pro Tyr Lys Met Glu His Phe Arg Trp Gly Ser Pro Pro Lys

         630         640         650         660         670         680         690
GAC AAA CGC TAC GGC GGT TTC ATG ACC TCG GAA AAA AGC CAA ACG CCG CTG GTC ACC CTG TTC AAA AAC
--- --- --- --- --- --- --- --- --- --- --- --- --- --- --- --- --- --- --- --- --- --- ---
Asp Lys Arg Tyr Gly Gly Phe Met Thr Ser Glu Lys Ser Gln Thr Pro Leu Val Thr Leu Phe Lys Asn

           700         710         720   726
GCT ATC GTC AAA AAT GCC CAC AAA AAA GGT CAA TAA
--- --- --- --- --- --- --- --- --- --- --- ---
Ala Ile Val Lys Asn Ala His Lys Lys Gly Gln stop
```

Figure S4. The cDNA sequence of porcine POMC used in this study.
